# Spatial pattern and associated factors of HIV testing and counselling among youths (15–24 years) in Ethiopia

**DOI:** 10.1186/s12889-021-10677-0

**Published:** 2021-04-01

**Authors:** Adugnaw Zeleke Alem, Alemneh Mekuriaw Liyew, Habtamu Alganeh Guadie

**Affiliations:** 1 Department of Epidemiology and Biostatistics, Institute of Public Health, College of Medicine and Health Sciences, University of Gondar, Gondar, Ethiopia; 2 School of Public Health, College of Medicine and Health Sciences, Bahir Dar University, Bahir Dar, Ethiopia

**Keywords:** HTC uptake, Spatial pattern, Ethiopia

## Abstract

**Background:**

HIV testing and counseling (HTC) services are key for HIV prevention, treatment, care, and support. Although the prevalence of HIV infection is high among adolescents and young adults, evidence suggests the utilization of HTC service among youth is very low in Ethiopia. Identifying factors and the geographic variation of HTC uptake is important to prioritize and design targeted prevention programs to increase its utilization and reduce HIV infection in hot spot areas.

**Methods:**

Data from the 2016 Ethiopian Demographic and Health Survey were used to analyze 10,781 youth aged 15–24 years. The spatial analysis was performed in ArcGIS 10.1. The Bernoulli model was used by applying Kulldorff methods using the SaTScan software to analyze the purely spatial clusters of HTC uptake. A multilevel logistic regression analysis was used to identify the associated individual and community-level factors of HTC uptake and estimate between community variance. All models were fitted in Stata version 14.0 and finally, the adjusted odds ratio (AOR) with a corresponding 95% confidence interval (CI) were reported.

**Results:**

In this study, the spatial patterns of HTC uptake were found to be non-random (Global Moran’s I = 0.074, *p* value< 0.001). Forty-seven primary clusters were identified that were located in the entire Somali region with a relative likelihood of 1.50 and the Log-Likelihood Ratio of 135.57. Youth who were ever married (AOR = 4.65; 95% CI; 4.05, 5.34), those attended higher education (AOR = 3.97; 95% CI; 3.10,5.08), those from richest household (AOR = 1.86; 95% CI; 1.44, 2.39), aged 20–24 years (AOR = 2.25; 95% CI; 2.02,2.51), having compressive HIV related knowledge (AOR = 2.05; 95% CI; 1.75,2.41), and exposed to media (AOR = 1.38; 95% CI; 1.22,1.57) were positive association with HTC uptake. However, being male (AOR = 0.81; 95% CI; 0.73,0.91) and having high HIV related stigma (AOR = 0.53; 95% CI; 0.42,0.67) were negatively associated with HTC uptake. At the community-level, youth from communities with a high percentage of educated (AOR = 1.45; 95% CI; 1.17,1.80) were more likely to utilize HTC compared with those from communities with low percentages of educated.

**Conclusion:**

The current study indicated differences in HTC uptake in the country. Both individual and community-level factors affected HTC uptake in Ethiopia. Multifaceted intervention approaches that consider individual and community factors are required to improve HTC uptake.

## Background

Human Immunodeficiency Virus (HIV) infection remains a major public health and medical concern throughout the world, especially it is more severe in Sub-Saharan Africa (SSA) [1]. There were 37.9 million people living with HIV at the end of 2018 globally. Of the global prevalence of HIV, Sub-Saharan constitutes nearly two-thirds of cases [2, 3]. Among People Living with HIV (PLHIV), 30% were young people aged 15 to 24 years [4]. Although the overall number of AIDS-related deaths decreased globally by 48% between 2005 and 2017, AIDS-related deaths among all adolescents and young people increased by 50% [5].

To end AIDS by 2030, the United Nations Program on HIV/AIDS (UNAIDS) has adopted the 90–90–90 strategic framework in 2014 [6]. This framework states that 90% of PLHIV know their status (diagnosed), 90% of those diagnosed receiving treatment, and 90% of those receiving treatment being virally suppressed by 2020 [7]. Increasing HIV testing and counseling (HTC) uptake is critical to achieving this target [8]. However, PLHIV who are aware of their HIV status (target one—90% of all HIV-positive people diagnosed) were low globally, which varied from 87% in the Netherlands to 11% in Yemen, making it more difficult to prevent the spread of the infection [9–12].

HTC services are key for HIV prevention, treatment, care, and support [13, 14]. It creates an opportunity for individuals linked to HIV care and treatment. Among the benefits of early linking patients in HIV care and treatment are reducing mother-to-child transmission, preventing uninfected partners from becoming infected, improving the quality of life, reducing morbidity and mortality related to opportunistic infections, and reducing the frequency of hospitalizations [15]. Knowing one’s HIV status may influence change in personal behavior, a critical part of efforts to prevent HIV. Moreover, through HTC, more people can get accurate information on HIV transmission, have a better understanding of their own risk or vulnerability to HIV infection, and HIV test result opens the door to accessing the range of HIV prevention options to stay themselves and their loved one’s HIV-free [16].

Ethiopia has developed and implemented different HIV-related programmes, aimed at youth over the past year to support the achievement of the 90–90-90 targets [17]. Besides, The Ethiopian government planned to end the epidemic at the end of 2030 with the expansion of HTC as a gateway for treatment and as a prevention strategy [18, 19]. Although the likelihood of acquiring the HIV infection is high among youths in Ethiopia because of the strong influence of peer pressure and engagement in unsafe sex, HTC uptake among young aged 15 to 24 years remained considerably low [20].

Several factors associated with HTC have been identified in different parts of the world. Most importantly, age [21–26], sex [22, 27, 28], educational status [22, 23, 29–32], marital status [21, 33, 34], socioeconomic status [21–23, 29], media exposure [29], having risk sexual behavior [21, 22, 29], having good knowledge about HIV [23, 29, 31], knowing where to get HTC [35], living in communities with higher education and wealth index [29], place of residence, having HIV-related stigma [21–23, 31, 35], religion [21, 29], and distance from a health facility [22] are significantly associated with HTC. However, inconsistent results have been reported in different settings in sub-Saharan Africa. For example, a study conducted in Ethiopia reported that being female is associated with lower odds of HTC [22], while a study conducted in Nigeria and sub-Saharan Africa countries found that being female is associated with higher odds of HTC [27, 28].

Studies have demonstrated that the spatial clustering of HIV prevalence in Ethiopia [17, 36]. These identified significant spatial clustering could be accounted for by different utilization of HTC across regions in Ethiopia. A study done in Nigeria reported regional variation of HTC among young aged 15–24 years [26]. Understanding the spatial pattern in HTC service utilization will help to design more specific programs to promote access and use of HTC services by young people in hot spot areas. However, previous studies conducted in Ethiopia investigated the prevalence and associated factors of HTC [20–22, 37], while all of these studies have not tried to explore the spatial distribution of HTC in Ethiopia among youths who are at higher risk for HIV acquisition.

Even though HTC uptake depends on the individual-level factors as well as community-level factors, previous studies have highlighted a range of individual factors associated with HTC uptake and there is a paucity of information on community-level factors that determines HTC uptake among young at the national level. Since young people are more vulnerable to HIV infection (youths aged 15–24 years are at high likelihood of the HIV/AIDS epidemic) [38] and the most productive segment of the population that forms the basic education sector which is vital to the creation of human capital [39], the current study tried to give special attention to HTC uptake among young aged 15–24 years. Focusing on this age group in the promotion of HTC is integral to achieving a 90–90-90 target [40]. Even though one study on HTC among young females is based on the nationally representative Ethiopian Demographic and Health Survey (EDHS) data [20], this study was failed to taking into account the clustering effect of EDHS data and the data they used were not weighted data. Moreover, to our knowledge, there is no study that addresses the spatial pattern of HTC uptake in Ethiopia. Identifying the geographic variation of HTC uptake is important to prioritize and design targeted prevention programs to increase its utilization and reduce HIV infection in hot spot areas. Therefore, this study will help policymakers to design and implement evidence-based interventions to the problem just by giving insight into the spatial pattern and risk factors associated with HTC uptake.

## Methods

### Study area and design

The study was conducted in Ethiopia. Ethiopia is a Sub-Saharan African country with an estimated population of 100,613,986 which makes it the second-most populous country in Africa. Administratively, Ethiopia is federally decentralized into nine regions and two city administrations and regions are divided into 68 zones. Also zones are divided into 817 administrative units called districts and then each district is further subdivided into 16,253 kebeles which is the lowest administrative unit. The present study used data from the EDHS, which is a nationally representative cross-sectional study conducted from January 18 to June 27, 2016.

### Data source and sampling techniques

This study utilizes a national-level data set generated from the 2016 Ethiopian Demographic and Health Surveys. The data are based on nationally representative surveys of 15–59 years (male) and women in their reproductive age (15–49 years), who were either permanent residents of the selected households or visitors who stayed in the household the night before the survey. The 2016 EDHS used five questionnaires such as the household questionnaire, the woman’s questionnaire, the man’s questionnaire, the biomarker questionnaire, and the health facility questionnaire. We used a woman’s questionnaire that was used to collect information from all eligible women age 15–49. The 2016 EDHS used the DHS Program’s standard Demographic and Health Survey questionnaires and the questionnaires were pretested in clusters surrounding Bishoftu that were not included in the 2016 EDHS sample. The questionnaire was first developed in English and then translated into Amarigna, Tigrigna, and Oromiffa language for appropriateness in approaching the study participants.

The Ethiopian Population and Housing Census (PHC), which was conducted in 2007 by the Ethiopia Central Statistical Agency was used as the sampling frame for the selection of the sampling units. The frame was a complete list of 84,915 enumeration areas (EAs) in which each EAs covers an average of 181 households. The EDHS employed a two-stage stratified cluster sampling technique based on census enumeration areas (EAs) and household samples. The first stage was the selection of EAs (645) in both rural (443) and urban (202) areas with probability proportional to the size, and the second stage involved households from a household list in the selected EAs. For this study, we limited our sample to youths aged 15–24 years. The samples for the final analysis after weighting was 10,781 (female = 6256 and male = 4525).

### Variables of the study

#### Dependent variable

The dependent variable for this study was HTC uptake, which was measured by asking the question: ‘Have you ever tested for HIV? This was a binary outcome variable coded 0 as “No” and 1 as “Yes”.

#### Independent variables

Based on previous literature [21–32, 34, 35], the independent variables for this study were group together into two broad categories: individual and community-level variables. Individual-level variables included in analysis were the following: sex, age, marital status, educational status, religion, wealth index, exposure to media (exposed to either of newspaper, radio, television or internet at least less than once a week) was categorized as “yes” and exposed to neither of newspaper, radio, television, and the internet was categorized as “no”), HIV/AIDS-related knowledge, HIV/AIDS-related stigma towards PLHIV and risky sexual behavior. HIV/AIDS-related knowledge was assessed by creating an index of correct responses to six questions included in the EDHS questionnaires, which are related to HIV prevention and misconceptions. The score was obtained by giving one point to respondents who knew the correct response and 0 to those who answered the incorrect response. Participants were classified as having low HIV related knowledge if they correctly answer ≤3 questions, having high knowledge if they correctly answer 4–5, and comprehensive knowledge if they correctly answer 6 questions. Stigma/discriminatory attitude towards PLHIV was measured using a set of six questions. Similarly, stigma towards PLHIV was categorized as “no stigma” (score 6), “low stigma” (score 4–5), “moderate stigma” (score 2–3), and “high stigma” (score ≤ 1). Finally, risky sexual behavior was assessed using a set of five questions and categorized as “no risk” (score 0), “some risk” (score 1), and “high risk” (score ≥ 2) [41].

The community-level variables were: a place of residence, distance from a health facility which was a self-reported response by respondents as a big problem or not a big problem. Some variables were obtained by aggregating individual-level variables into community-level variables. These variables include community education (aggregate values of education measured by the proportion of youths with a minimum of primary level of education derived from data on the level of education), community poverty level (proportion of youth in the poorest and poorer quintile derived from data on wealth index), community-level of media exposure (proportion of youth exposed to at least one type of media; radio, newspaper television, and internet), community HIV/AIDS-related knowledge (the proportion of youth with a minimum of high HIV related knowledge in the community), and HIV-related stigma (percentage of youths with accepting attitudes towards people living with AIDS). Each aggregated community variable was categorized into low and high on the basis of the national median value since they were not normally distributed.

### Data analysis procedure

#### Spatial analysis

Longitude and latitude data having 0 was dropped and a total of 622 clusters were included in spatial analysis. ArcGIS 10.1 was used for the analysis. The spatial autocorrelation (Global Moran’s I) statistic measure was used to evaluate whether the HTC uptake patterns are dispersed, clustered, or randomly distributed in the study area. Moran’s I is a spatial statistics used to measure spatial autocorrelation by taking the entire data set and produce a single output value that ranges from − 1 to + 1. A positive value for Moran’s Index indicates a clustered pattern of HTC uptake, while a negative value for Moran’s Index indicates a dispersed pattern and HTC uptake distributed randomly if I value is zero [42, 43]**.**

To identify and detect clusters of HTC uptake in the study area, a spatial scan statistic was employed to adjust for the underlying populations in each survey cluster using Kuldorff’s SaTScan version 9.6 program. Spatial scan statistical analysis was used to classify statistically important HTC uptake hotspots areas. The spatial scan method uses a circular window that moves across the map and at each position; the radius of the circular window varies repeatedly from zero up to a set maximum radius of 50 which restricts the maximum size of the window from exceeding 50% of the total study population. In this study, youths who are not ever tested for HIV were taken as cases whereas youths who ever tested for HIV were taken as controls to fit the Bernoulli model. The numbers of cases in each location have Bernoulli distribution and a maximum spatial cluster size of < 50% of the population was used as an upper limit. Z-score is computed to determine the statistical significance of clustering, and the *p*-value was used to determine if the number of observed cases within the potential cluster was significant or not. The null hypothesis of no clusters was rejected when the *p*-value ≤0.05 [44, 45].

The spatial interpolation technique was applied to predict the magnitude of HTC uptake on the unsampled areas based on the values observed on the sampled EAs. The magnitude of HTC uptake distribution was determined using the ordinary Kriging method of interpolation in ArcGIS. This is a deterministic interpolation model that assigns values to locations where no measurements have been taken, based on how far those locations are to sentinel locations where measurements have been taken [46].

#### Multi-level analysis

The data were downloaded and coded using Stata version 14 and checked for completeness before doing any statistical analysis. Descriptive data analysis was conducted on all variables of interest using percentages. Due to the non-proportional allocation of the sample to different regions and their urban and rural areas as well as the possible differences in response rates, data were weighted using sampling weight before any statistical analysis to account for the sampling design. First, the bivariable analysis was conducted to assess the associations between the outcome variable and the independent variables. All variables with a *p*-value < 0.2 in the bi-variable analysis were included in the multivariable analysis. The Adjusted Odds Ratio (AOR) with a 95% confidence interval (CI) was reported to show the strength of association.

A two-level multivariable logistic regression analysis was used to estimate the effect of independent variables on the outcome variable while accounting for the hierarchical structure of the DHS data to get a reliable standard error and made the appropriate inference. The model fitting process involved four stages of estimation. The first model (model I) was an empty model without any explanatory variables, to calculate the extent of cluster variation on HTC uptake. The variation between cluster (EAs) were assessed by computing Intra-class Correlation Coefficient (ICC), a Proportional Change in Variance (PCV), and Median Odds Ratio (MOR). The ICC and MOR were computed as follows; ICC=VA/ (VA + 3.29) and MOR = exp. [√(2 × VA) × 0.6745]. Where V_A_ is the area/cluster level variance and 0.6745 is the value from the 75th percentile of the cumulative distribution function of the normal distribution with mean = 0 and variance = 1 [47, 48]. Additionally, PCV was calculated as; PCV = (VA-VB)/VA)*100, where; VA is community variance of the model without covariates (model 1) and VB is community variance in the models with more covariates (model 2, model 3, or model 4) [48]. The second model (model II) was adjusted with individual-level variables and the third model (model III) was adjusted for community-level variables. Finally, Model IV included both individual and community-level variables was performed. Then the parsimonious model was chosen by using deviance.

## Results

### Background characteristics of the study participants

A total of 10,781 young aged 15–24 years were included in the analysis. The majority (58.0%) of the respondents were females. More than two-thirds (69.5%) of the respondents were rural residents. Forty-one percent of the respondents were Orthodox in terms of religion. More than half (52.6%) of the respondents attended primary education and a majority (73.8%) of respondents were ever married (Table 1).

**Table 1:** Frequency distribution of background characteristics of the study participants

Variables	Frequency	Percent
Sex		
Female	6256	58.0
Male	4524	42.0
Age of respondent		
15 to 19	5964	55.3
20 to 24	4817	44.7
Educational status		
No education	1879	17.4
Primary	5673	52.6
Secondary	2395	22.2
Higher	834	7.8
Religion		
Orthodox	4414	40.9
Muslim	4183	38.8
Protestant	1994	18.5
Others	190	1.8
Wealth index		
Poorest	2389	22.2
Poorer	1563	14.5
Middle	1564	14.5
Richer	1670	15.5
Richest	3595	33.3
Residence		
Rural	7493	69.5
Urban	3288	30.5
Marital status		
Never married	7434	26.2
Ever married	20,937	73.8
Media exposure		
No	4351	40.4
Yes	6430	59.6
Age at first sex		
< 20	7900	73.3
≥ 20	2881	26.7
Distance to health facility		
Big problem	2553	46.1
Not big problem	2988	53.9
Know a place to get HIV test		
No	2102	20.8
Yes	7980	79.2
Chewed khat		
No	8775	81.6
Yes	1972	18.4
Ever drunk alcohol		
No	6561	61.0
Yes	4186	39.0

### HIV/AIDS-related knowledge, HIV/AIDS-related stigma and risky sexual behavior

Three-fourth (75.3%) of respondents knew that regular condom usage during sex reduces the likelihood of getting HIV. The majority (85.5%) of respondents believed that HIV infection is not transmitted by sharing food with a person who has AIDS. Nearly half (48.6%) of respondents have high HIV related knowledge. Nearly two-thirds (63.3%) of participants believed that children with HIV should be allowed to attend school together with children without HIV. The majority (43.4%) of respondents had low HIV related stigma (Table 2).

**Table 2:** Frequency distribution of HIV/AIDS-related knowledge, HIV/AIDS-related stigma and risky sexual behavior among young aged 15–24 years, E*thiopian Demographic and Health Survey* 2016

Variables	Frequency	Percent
**Knowledge Indicators**		
Always use condoms during sex		
No	2487	24.7
Yes	7595	75.3
Have 1 sex partner only, who has no other partners		
No	6567	24.8
Yes	19,970	75.2
Can get HIV from mosquito bites		
No	6198	61.5
Yes	3884	38.5
Can get HIV by sharing food with person who has AIDS		
No	8640	85.7
Yes	1442	14.3
Can get HIV by witchcraft or supernatural means		
No	8560	84.9
Yes	1522	15.1
A healthy looking person can have HIV		
No	3090	30.6
Yes	6992	69.3
Overall knowledge		
Low	2295	22.8
High	4899	48.6
Comprehensive	2888	28.6
**Stigma Indicators**		
Would be ashamed if someone in the family had HIV		
No	6386	63.3
Yes	3696	36.7
Would buy vegetables from vendor with HIV		
No	4508	44.7
Yes	5574	55.3
Children with HIV should be allowed to attend school with children without HIV		
No	3701	36.7
Yes	6381	63.3
People hesitate to take HIV test because reaction of other people if positive		
No	2654	26.3
Yes	7427	73.7
People talk badly about people with or believed to have HIV		
No	4128	41.0
Yes	5954	59.0
People with or believed to have HIV lose respect from other people		
No	4500	44.6
Yes	5582	55.4
Overall stigma		
No	1929	19.1
Low	4369	43.4
Moderate	2853	28.3
High	930	9.2
**Risky Sexual Behavior Indicators**		
Had any STI in last 12 months		
No	10,035	99.5
Yes	47	0.5
Had genital sore/ulcer in last 12 months		
No	10,025	99.4
Yes	56	0.6
Had genital discharge in last 12 months		
No	9991	99.1
Yes	91	0.9
Had multiple life time sexual partner		
No	3056	75.1

### Spatial pattern of HTC uptake

In this study, the spatial patterns of HTC uptake were found to be non-random. The global spatial autocorrelation analysis revealed a clustering pattern of HTC uptake among youths across Ethiopia (Global Moran’s I = 0.074, *p* value< 0.001).

### Interpolated proportion of HTC uptake

**Fig. 1:**
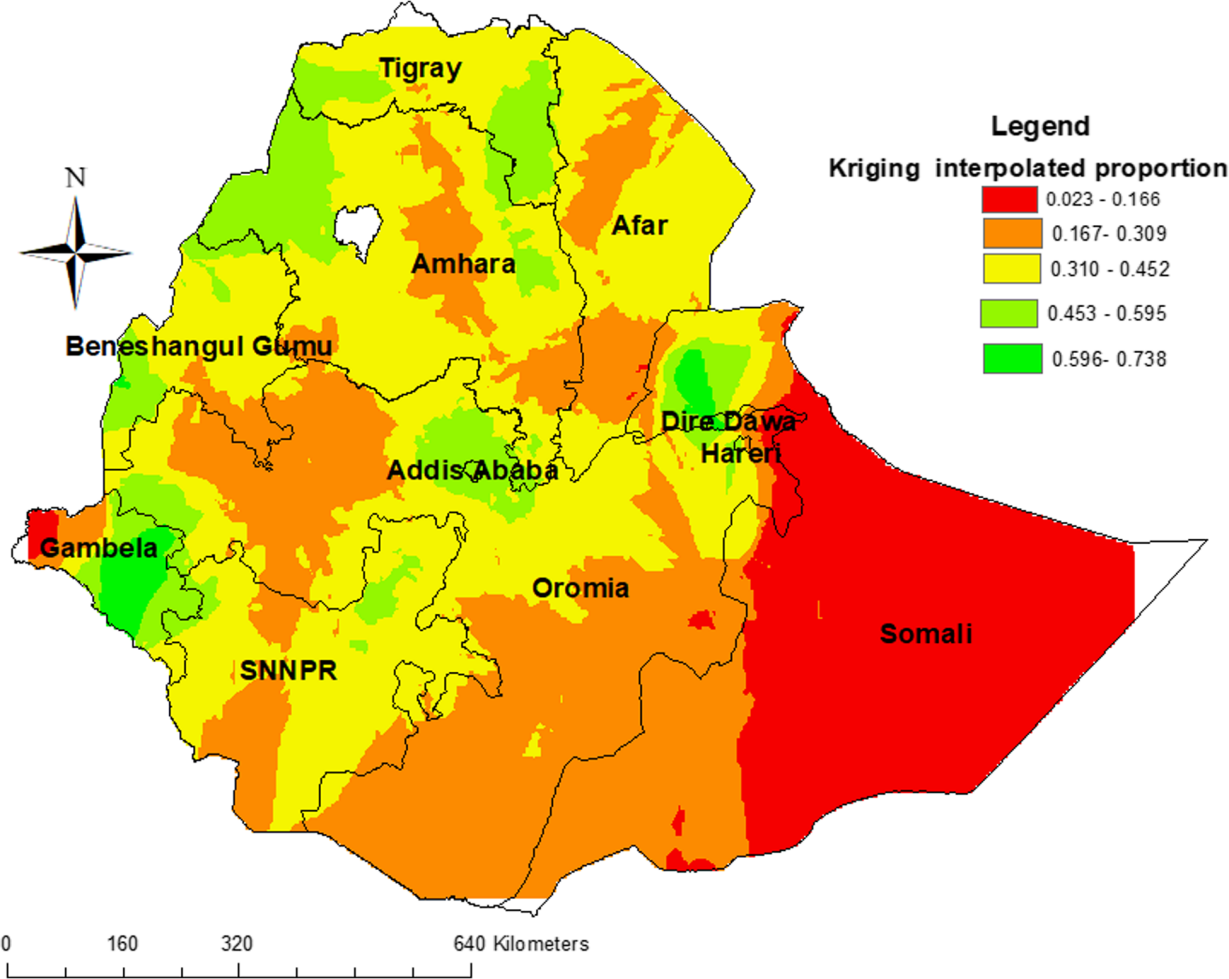
Kriging interpolation of HIV testing and counseling uptake among youths in Ethiopia Using Arc-GIS version 10.6 (Source: Shape file from Central Statistical Agency, Ethiopia, 2013)

The predicted HTC uptake over the area increases from red to green -colored areas. The red color indicates high-risk (low HTC utilization) areas of predicted HTC uptake and the green color indicates the predicted low-risk areas of HTC uptake. The kriging prediction map with red color told us that the entire Somali and western parts of the Gambela regions of the nation were predicted as risk areas for HTC uptake. While highest HTC uptake rates were detected in Addis Ababa, Dire Dawa, the western part of Amhara, some southern parts of Gambela, and southern parts of Tigray (Fig. 1).

### Spatial scan statistical analysis

**Fig. 2:**
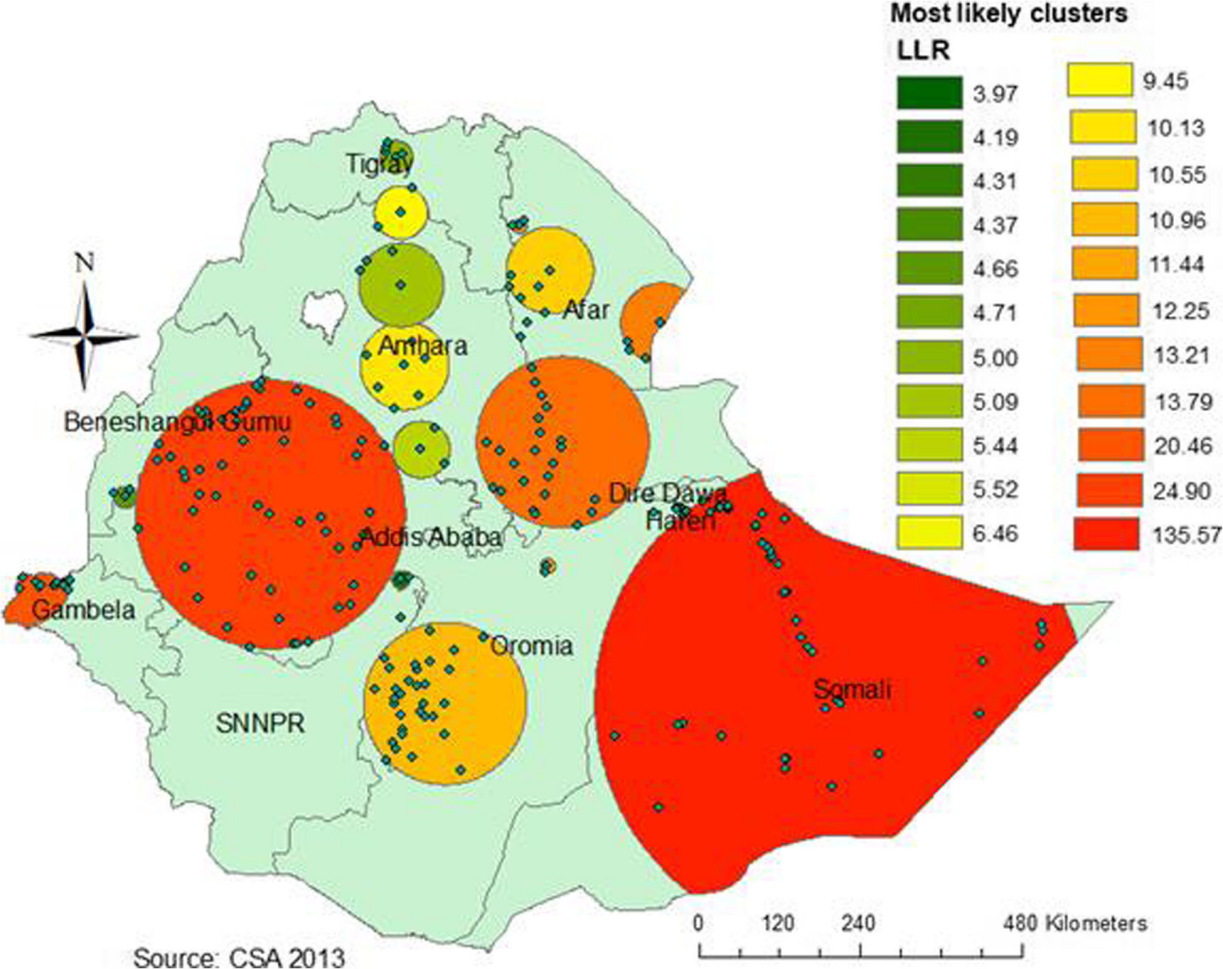
Sat Scan analysis of HTC uptake using *the Kuldorff’s SatScan approach* among youths in Ethiopia (Source: Shape file from Central Statistical Agency, Ethiopia, 2013)

In the study, about 193 significant clusters were recognized. Of these 47 clusters were primary clusters that were located in the entire Somali region (Fig. 2). This spatial window was centered at 6.745502 N, 44.259011 E with a 362.27 km radius, with a relative likelihood of 1.50 and the Log-Likelihood Ratio (LLR) of 135.57, at *p*-value < 0.001. It indicated that youths found inside the window were 1.50 times riskier for HTC utilization as compared with those found outside the window. In addition, the remaining spatial windows were secondary clusters (Fig. 2).

### Factors associated with HTC utilization

#### Random effects and model comparison

The ICC in the null model indicated that 23% of the total variability for HTC uptake was due to differences between clusters/communities. Regarding PCV, about 58.6% of the variability in HTC uptake was explained by the full model (model IV). The highest PCV in model IV indicates including the community-level variables has improved the model. The median odds ratio also revealed that HTC uptake among youth was heterogeneous among clusters. In the null model, MOR was found 2.57, this means if we randomly select households from different clusters, households at the cluster with higher HTC uptake had 2.57 times higher odds of HTC uptake as compared with those households at cluster with lower HTC uptake. It was decreased from 2.57 in the null model to 1.84 in the final model (model IV), which indicates the model IV explains the low variability of HTC uptake (Table 3).

**Table 3:** Random effect and model fitness of HTC uptake among youth aged 15–24

Random measures	Model I	Model II	Model III	Model IV
Community-level variance	0.99	0.44	0. 49	0.41
ICC	0.23	0.12	0.13	0.11
MOR	2.57	1.88	1.94	1.84
PCV(%)	Reference	55.5	50.5	58.6
**Model fitness**				
Deviance (−2LL)	13,646.09	11,104.12	13,271.36	11,020.63

### Fixed effects

In the multilevel logistic regression analysis, both individual-level and community level-level were significantly associated with HTC utilization. Among variables included in multivariable analysis sex, age, educational status, marital status, wealth index, HIV related knowledge, HIV related stigma, media exposure and community education were statistically significantly associated variables.

The odds of HTC uptake among young aged 15–24 years who were ever married was 4.65 (AOR 4.65; 95% CI; 4.05, 5.34) times higher compared to never married. The odds of HTC uptake among young aged 15–24 years who attended primary, secondary, higher education was 1.66 (AOR 1.66; 95% CI; 1.40, 1.96), 2.88 (AOR 2.88; 95% CI; 2.36, 3.51), and 3.97 (AOR 3.97; (95% CI; 3.10,5.08) times higher compared to those who did not attend formal education respectively. The odds of HTC uptake among poorer, middle, richer, richest was 1.28 (AOR 1.28; 95% CI; 1.06, 1.56), 1.28 (AOR 1.26; 95% CI; 1.03, 1.55), 1.45 (AOR 1.45; 95% CI; 1.18,1.79), and 1.86 (AOR 1.86; 95% CI; 1.44, 2.39) times higher as compared to poorest respectively. Similarly, being aged 20–24 years (AOR 2.25; 95% CI; 2.02,2.51), having high HIV related knowledge (AOR 1.75; 95% CI; 1.52,2.02) and compressive knowledge (AOR 2.05; 95% CI; 1.75,2.41) and exposed media (AOR 1.38; 95% CI; 1.22,1.57) had positive association with HTC uptake.

However, being male and HTC uptake was found to be negatively associated (AOR 0.81; 95% CI; 0.73,0.91). similarly, having low, moderate and high HIV related stigma was (AOR 0.83; 95% CI; 0.73,0.95), (AOR 0.66; 95% CI; 0.57,0.77), (AOR 0.53; 95% CI; 0.42,0.67) negatively associated with HTC uptake as compared with those who haven’t HIV related stigma respectively.

Moreover, the study revealed that the odds of HTC uptake was higher among community with more educated youths (AOR 1.45; 95% CI; 1.17,1.80) compared to those living in communities with low educated youths (Table 4).

**Table 4:** Multilevel analysis of factors of HTC uptake among young aged 15–24 years in Ethiopia, 2016

Variables	COR (95% CI)	AOR (95% CI)
**Sex**		
Female		1
Male	0.72 (0.66–0.78)	0.81 (0.73–0.91)*
**Age**		
15–19	1	1
20–24	3.69 (3.36–4.04)	2.25 (2.02–2.51)*
**Marital status**		
Never married	1	1
Ever married	4.34 (3.88–3.85)	4.65 (4.05–5.34)*
**Religion**		
Orthodox	1	1
Protestant	0.74 (0.63–0.87)	0.91 (0.77–1.08)
Muslim	0.53 (0.46–0.61)	0.78 (0.68–1.02)
others	0.50 (0.33–0.75)	0.79 (0.51–1.24)
**Level of women education**		
No education	1	1
Primary	1.52 (1.32–1.75)	1.66 (1.40–1.96)*
Secondary	3.40 (2.90–3.99)	2.88 (2.36–3.51)*
Higher	7.27 (5.90–8.95)	3.97 (3.10–5.08)*
**Wealth index**		
Poorest	1	1
Poorer	1.79 (1.50–2.13)	1.28 (1.06–1.56)*
Middle	1.87 (1.57–2.23)	1.26 (1.03–1.55)*
Richer	2.39 (2.01–2.85)	1.45 (1.18–1.79)*
Richest	4.67 (3.97–5.51)	1.86 (1.44–2.39)*
**Knowledge**		
Low	1	1
High	2.09 (1.85–2.36)	1.75 (1.52–2.02)*
Comprehensive	2.62 (2.29–3.00)	2.05 (1.75–2.41)*
**Stigma**		
No	1	1
Low	0.78 (0.69–0.87)	0.83 (0.73–0.95)*
Moderate	0.50 (0. 43–0.57)	0.66 (0.57–0.77)*
High	0.31 (0.26–0.38)	0.53 (0.42–0.67)*
Residence		
Urban	1	1
Rural	0.32 (0.27–0.37)	0.96 (0.73–1.23)
**Media exposure**		
No	1	1
Yes	1.96 (1.76–2.17)	1.38 (1.22–1.57)*
**Community poverty level**		
Low	1	1
High	0.32 (0. 27–0.37)	0.82 (0.66–1.01)
**Community education**		
Low	1	1
High	3.35 (2.80–3.99)	1.45 (1.17–1.80)*
**Community knowledge**		
Low	1	1
High	2.54 (2.15–3.00)	1.01 (0.67–1.53)
**Community stigma**		
Low	1	1
High	2.59 (2.20–3.06)	1.16 (0.76–1.80)
**Community media exposure**		
Low	1	1
High	0.33 (0. .28–0.39)	1.01 (0.83–1.23)

*P value < 0.05

## Discussion

This study examined the spatial distribution, individual, and community-level factors associated with HTC uptake in Ethiopia. It identified a spatial variation of HTC uptake in Ethiopia, with high risk (low HTC uptake) in the Somali region. Similarly, a study conducted in Nigeria suggested that regional variation of HTC among youths [26]. This spatial variation of HTC uptake could be explained due to the spatial clustering of HIV infection in the country [17, 36, 49]. The detection of low HTC uptake rates in the Somali region implies that HIV-related knowledge and education are likely to be suboptimal in the Somali region [50, 51]. This can be also related to the fact that the effectiveness of implementing different HIV-related programmes, including the expansion of HTC uptake highly depends on the strength of local implementers’ inappropriately utilizing resources and implementing interventions to the local context since most of the HIV intervention programs are donor-driven. Furthermore, these variations possibly explained due to the wide regional difference in educational status, media exposure culture, and living conditions. For example, in this study, 36.1% of youths in the Somali region were not attained formal education compared with 2.8% of youths in Addis Ababa. Moreover, in this study, only 36.8% of youths in the Somali region have media exposure, which is lower than other regional states of Ethiopia, while nearly all (98.2%) of youths in Addis Ababa have media exposure. These low educational status and media exposure of the Somali region might lead to low utilization of HTC. This is because low education level and low media exposure are usually associated with low utilization of HTC [22, 23, 29–32]. Therefore, this study suggests that more attention is needed in the region within the cluster windows.

In our study, being male decreases the odds of HTC uptake, which is supported by a study done in Nigeria [27], Uganda [52], Tanzania [53], Kenya [54], South Africa [25], and among four Sub-Saharan Africa countries [28]. This might be due to the WHO recommends universal HIV testing for all pregnant women and prompt treatment among HIV-positive women in order to prevent mother-to-child transmission of HIV [55]. Also, the Ethiopian government started routine HIV testing and counseling as well as the integration of HIV counseling and testing with family planning and maternal, newborn, and child health services [56]. Thus, adoption of universal HIV testing for all pregnant women and integration of HIV counseling and testing with family planning and maternal, newborn and child health creates an opportunity for HIV testing during maternal health care utilization among women [57], whereas men are reluctant to come to the maternal health services clinic with their wives to be tested [58, 59]. HTC places more emphasis on autonomy than routine testing. Men in many African countries including Ethiopia are the key decision-makers at home, in workplaces, in parliament, and in religious institutions. This indicates HTC services may be improved through increased uptake of testing among men. Therefore, the government and WHO could play a more positive role for all people not only for women, to better utilize HTC through such kinds of counseling and testing promotions.

However, this finding is argued with a previous study conducted in Ethiopia which showed that being male was strongly correlated with HTC uptake [22]. This disagreement might be due to the differences in the study population. Unlike the current study, which was conducted on both rural and urban populations (nationally representative data), the previous study was limited to the rural population.

It is to be noted that the odds of HTC uptake were highest among younger people aged 20–24 years. This finding was similar to studies done in Ethiopia [21, 22], Uganda [24], South Africa [25], and Nigeria [23, 26]. This finding could be explained youth aged 15–19 have a lower self-perceived likelihood of HIV [41]. Moreover, this could be due to the fact that youth aged 20–24 are more likely to be sexually active, more likely to be married, and more likely to be economically empowered than youth aged 15–19.

This study revealed higher utilization of HTC uptake among youth with higher educational levels, an association that had been reported in other studies conducted in Ethiopia [22], Nigeria [23, 32], Zambia [30], Burkina Faso [29] and Malawi [31]. A possible explanation for this result could be that as the educational status of youth improves they would have more awareness about HTC services, understanding of health-related information, and hence HTC uptake increases [60]. For example, education provides more opportunities to clearly understand HIV infection and prevention. Education also empowers one’s own autonomy to make decisions to visit the health facility and use health services [61, 62]. Therefore, Ethiopian government should be strengthening the available initiatives to enable youths to attend formal education to improve HTC uptake.

In the current analysis, we also found that ever-married youths were more likely to have HTC uptake compared with never-married. This result is in line with studies done in Ethiopia [21], three Caribbean countries [34], and China [35]. This finding could result from the majority of people believe that HTC is useful for preparing for marriage [32] and different organizations like UNAIDS and religious groups promote essential counseling and testing for couples intending to get married. Furthermore, the Ethiopia Federal Ministry of Health encourages couples to learn about their HIV status and make informed decisions about their future that might have played a positive role for married people to better utilize HTC. Also, it is possible that married women are more likely to get the perinatal HIV testing service available in most health institutions, which might also contribute to the improvement of HTC uptake.

Consistent with a study from Burkina Faso [29], youths exposed to mass media have higher odds of HTC uptake. The possible reasons for this finding might be due to the mass media frequently cover health-related topics, are the leading source of information about important health issues and exposure to it provides increased awareness and knowledge. Additionally, exposure to mass media influences behaviors, attitudes, and social norms that may positively affect the use of health services [63]. Besides, in fact, mass media are an effective measure to reach populations on a large scale and increase the use of health services. Therefore, it should be considered as possible strategies for health promotion by health-care providers and policy-makers to increase HTC services in Ethiopia.

In line with previous studies [21–23, 29, 35, 41], this study revealed that the youth belonging to wealthier households have higher odds of HTC uptake than the youth of the poorest households. The possible reason might be socio-economic position affect utilization HTC service uptake in many aspects, even in countries with universal health care system [64, 65]. In addition, youth at higher socio-economic positions are more likely to have a higher educational level and economically advantaged to seek and access HTC uptake services than those at a lower socioeconomic position.

Our study found that also HIV-related knowledge was associated with HTC uptake. This is in agreement with different studies done in Ethiopia [21], Nigeria [23], Burkina Faso [29], and Malawi [31]. Increased HIV related knowledge may reduce HIV related stigma and discrimination [66, 67]. To improve the uptake of HTC among youth, it is important to equip them with information about HIV. This result highlighted the importance of HIV related knowledge to the increment of HTC uptake. Unfortunately, different studies indicate the level of HIV knowledge among youth in different countries including Ethiopia is very low [68–71].

In the current study, those individuals who have HIV related stigma had lower odds of HTC uptake. This finding is supported by studies conducted in Ethiopia [21, 22, 41], Nigeria [23], Malawi [31], and China [35]. HIV/AIDS remains the most stigmatized disease globally and stigma continues to be a major barrier to seeking access to healthcare services including HTC uptake service [72, 73]. HIV-related stigma has been associated with a lack of proper information and awareness, fear, and many people think of HIV as a disease that only certain groups get that leads to negative moral judgment from those living with the disease [74]. Therefore, due to fear of stigmatization youth would not be willing to use HTC services and ultimately impose a negative impact on HTC uptake among youth.

Apart from significant individual-level factors associated with HTC uptake, the study also revealed significant community effects. Specifically, youth from communities with a high percentage of educated youth had higher odds of HTC uptake compared with those from communities with low percentages of youth. This is supported by a study done in Burkina Faso [29]. This is possible youths from communities with a high percentage of educated youth may learn from others on the importance of using HTC services and where these may be accessed. Furthermore, educated youth have better chances of understanding health messages and demand services.

### Strengths and limitations

The study included a nationally representative sample of youth in Ethiopia therefore, results from the current analysis may be generalized to Ethiopian youths. Unlike previously conducted studies that mainly focused on only individual-level factors, this study measured community-level factors. Additionally, the current study applied multilevel analysis to accommodate the hierarchical nature of the EDHS data in estimating the determinants factors with a combination of spatial analysis that allows an understanding of the geographic variation of HTC uptake. Despite the abovementioned strengths, the result of this study should be interpreted in light of the following limitations. First, since we used the cross-sectional nature of the study means causality cannot be inferred. Second, the SaTScan analysis detects only circular clusters, irregularly shaped clusters were not detected. Third, recall and social desirability biases might be a possible limitation because the DHS survey is relied on respondents’ self-report based on their memories and HTC is a sensitive issue. Finally, due to the secondary nature of data used important variables like health-care service factors such as cost, perceived quality, and accessibility, facility-related barriers to HTC use, and fear of HIV results were not assessed in this study.

## Conclusion

The current study indicated spatial variation in HTC uptake in the country. It identified spatial clusters of low HTC uptake in Somalia Region and Addis Ababa, Dire Dawa, the western part of Amhara, some southern parts of Gambela, and southern parts of Tigray with the highest utilization rate. Both individual and community- level factors affected HTC uptake in Ethiopia. Youth who were ever married, those attended education, those from richest household, aged 20–24 years, having HIV related knowledge, exposed to media, and youth from communities with a high percentage of educated were positive association with HTC uptake. However, being male and having high HIV related stigma were negatively associated with HTC uptake. Multifaceted intervention approaches that consider individual and community factors are required to improve HTC uptake. Additionally, targeted interventions are needed in those risky areas, especially focusing on the improvement of education, mass media, and wealth of household.

## Data Availability

All result based data were found in the manuscript and the datasets used and/or analyzed during the current study is available from http://www.dhsprogram.com.

## Abbreviations

AOR: Adjusted Odds Ratio
CI: Confidence Interval
EDHS: Ethiopian Demographic and Health Survey
HIV: Human Immunodeficiency Virus
HTC: HIV testing and counseling
ICC: Intra-class Correlation Coefficient
LLR: Log likelihood Ratio
MOR: Median Odds Ratio
PCV: Proportional Change in Variance
PLHIV: People Living with HIV
